# Toxoplasmosis as a cause of life‐threatening respiratory distress in a dog receiving immunosuppressive therapy

**DOI:** 10.1002/ccr3.2121

**Published:** 2019-04-02

**Authors:** Amy Pepper, Caroline Mansfield, Andrew Stent, Thurid Johnstone

**Affiliations:** ^1^ Faculty of Veterinary and Agricultural Sciences, Translational Research and Animal Clinical Trial Study Group (TRACTS), U‐Vet Animal Hospital Werribee The University of Melbourne Melbourne Victoria Australia

**Keywords:** ciclosporin (INN)/cyclosporine, dog, immune‐mediated disease, immunosuppression, prednisolone, toxoplasmosis

## Abstract

Disseminated toxoplasmosis is a potentially fatal complication in dogs receiving immunosuppressive therapy, particularly if multiple immunosuppressive drugs are used. Toxoplasmosis should be considered if signs of acute respiratory or hepatic disease develop, and diagnosis would rely on demonstration of organisms via cytology or PCR rather than a single time‐point serological assay.

## 1 INTRODUCTION

Toxoplasmosis was diagnosed in a dog presenting in respiratory distress; the dog was receiving immunosuppressive medication for immune‐mediated disease prior to the presentation. The dog was being treated for immune‐mediated hemolytic anemia with ciclosporin, azathioprine, and prednisolone. The diagnosis of toxoplasmosis was made via identification of tachyzoites in bronchoalveolar lavage fluid. Despite intensive treatment, the dog died shortly after diagnosis. Life‐threatening systemic toxoplasmosis secondary to immunosuppression is well documented in cats. The case described herein illustrates that toxoplasmosis should also be considered in dogs receiving immunosuppressive therapy, particularly when respiratory signs develop. Further studies are needed to evaluate the prevalence of toxoplasmosis in immunosuppressed dogs, whether certain drugs are associated with a higher risk of toxoplasmosis and whether screening for *Toxoplasma gondii*before or during administration of multi‐agent immunosuppressive treatment is beneficial.

*Toxoplasma gondii* is an intracellular coccidian belonging to the phylum Apicomplexa; its sexual life cycle completes in the domestic cat and other Felidae*,*which shed oocysts in their feces. Sporozoites develop in oocysts 1‐5 days after exposure to humidity and oxygen and are infectious to many warm‐blooded vertebrates, including dogs and humans. These species act as intermediate hosts, in which *T gondii* multiplies asexually by forming rapidly dividing, systemically spreading tachyzoites and essentially dormant bradyzoites that persist in nerve, muscle or visceral tissues.^1^ Routes of infection in dogs are via ingestion of feces containing oocysts, carnivorism and transplacental transmission. Uptake of oocyts with water, blood transfusion, or organ transplantation may be further potential sources of infection.^1^, ^2^, ^3^ *Toxoplasma gondii* is ubiquitous, with serological surveys in Australia and Ireland demonstrating exposure rates of up to 40% in dogs.^4^, ^5^ However, systemic toxoplasmosis in the dog is uncommonly reported, and most cases described to date were concurrently affected by diseases such as canine distemper.^6^, ^7^, ^8^, ^9^, ^10^ A potential link between immunosuppressive treatment and the occurrence of toxoplasmosis has so far been suggested in only two dogs.^3^, ^11^

Cell‐mediated immunity is the main defense against toxoplasmosis.^12^ An immune‐competent host is unlikely to develop systemic clinical toxoplasmosis, as induction of the T‐cell‐mediated immune response creates resistance to the tachyzoite stage. After development of immunity, tachyzoites are cleared from host tissues, but bradyzoites persist and infection becomes latent. A healthy immune response to *T gondii*is required to prevent the reactivation of latent infections. The reemergence of tachyzoites, and associated pathological changes, is therefore often observed in immunocompromised hosts.^8^, ^13^

This report describes a dog that received immunosuppressive treatment for immune‐mediated hemolytic anemia and was subsequently diagnosed with systemic toxoplasmosis.

## 2 CLINICAL FEATURES

A 16‐month‐old male neutered Border collie dog was diagnosed at the primary veterinary practice with immune‐mediated hemolytic anemia (IMHA) based on hemogram findings of a regenerative anemia with moderate to marked spherocytosis. The dog lived in an urban, tick‐free area and only travelled within the region; there was no recent vaccination or toxin exposure. Raw meat had been fed occasionally before the onset of disease but not since immunosuppressive treatment had begun. The dog had been treated with prednisolone (Pred‐X 20, Apex Laboratories Pty Ltd, Australia) with doses varying from 1.6 to 3.5 mg/kg/d PO depending on clinical signs and PCV, and with azathioprine (Imuran, GlaxoSmithKline, Australia) at 2 mg/kg/d PO since diagnosis. Prior to referral, the dog received a whole blood transfusion and omeprazole (2 mg/kg q 12 hours PO) was started. An abdominal ultrasound, thoracic radiographs, and activated partial thromboplastin time (APTT) were performed and were unremarkable.

Seven weeks after initial presentation, the dog was referred to the U‐Vet Animal Hospital Werribee for evaluation of persistent anemia despite the prednisolone and azathioprine treatment. On initial examination, the dog was bright and normothermic (38.8°C), and thoracic auscultation was unremarkable. Marked temporal muscle atrophy, a pendulous abdomen and poor hair regrowth from clipped sites were present, likely because of chronic corticosteroid treatment. After review of previous reports, blood results and thoracic radiograph images, hematology and serum biochemistry were performed, with relevant results reported in Table 1 (Day 1). A poorly regenerative anemia was present, with ongoing red cell destruction (polychromasia, sherocytosis). White cell and biochemical abnormalities were thought to reflect corticosteroid use. Ciclosporin (Atopica, Novartis Animal Health, Australasia; 9 mg/kg/d PO, divided into two doses) and aspirin (Compounded, BOVA, Australia; 0.5 mg/kg/d PO) were prescribed as an additional immunosuppressive drug and to reduce the risk of thromboembolic disease, respectively. Omeprazole was reduced to 0.9 mg/kg q 12 hours PO. The dog had a packed cell volume (PCV) of 0.24 L/L the following day and was discharged. The owner was advised not to feed any raw meat.

**Table 1:** Complete blood count (CBC) and serum biochemistry

Parameter	Results				Unit	Reference interval
Date	Day 1	Day 5	Day 9	Day 11		
PCV	**0.19**	**0.23**	**0.25**	**0.27**	L/L	0.37‐0.44
Red cell count	**2.5**	**2.7**	**3.0**	**3.3**	×10^12^/L	5.5‐8.5
Reticulocytes	1.5	**2.4**		**4.3**	%	0‐1.5
Platelets	**557**	**698**	415	206	×10^9^/L	200‐500
WBC	**29.0**	**41.5**	**26.4**	15.6	×10^9^/L	5.05‐16.76
Neut M	9.53	**38.6**	**24.2**	**12**	×10^9^/L	3‐11.5
Neut B	NR	NR	NR	**3.0**	×10^9^/L	0‐0.3
Lymphocytes	1.77	**0.4**	1.6	**0**	×10^9^/L	1‐3.6
Monocytes	**2.65**	**2.5**	0.6	0.6	×10^9^/L	0.16‐1.12
Eosinophils	0.02	NR	NR	NR	×10^9^/L	0.06‐1.23
Morphology	a, c, j, k	a, b, c, d, e,	a, c, d, g,	a, c, d, g, h, i		
Urea	**10.9**		**18.2**	**19.1**	mmol/L	2.5‐9.6
ALKP	**2445**			**2190**	iu/L	23‐212
ALT	**174**			**1322**	iu/L	10‐100
GGT	**64**			**171**	iu/L	0‐7
Total protein	63			**45**	g/L	54‐78
Glucose	5.45			**8.0**	mmol/L	3.3‐6.7
Cholesterol	6.98			**9.9**	mmol/L	3.9‐7.8
Bilirubin	3			**46**	µmol/L	0‐10

a, Anisocytosis; b, Target cells; c, Polychromasia; d, Howell‐Jolly bodies; e, Rouleaux formation; f, Poikilocytosis; g, Basophilic stippling; h, Schistocytosis; i, Toxic neutrophils (many); j, Spherocytes; k, Saline agglutination positive.

Bold indicates outside of reference range.

ALKP, alkaline phosphatase; ALT, alanine aminotransferase; B, Bands; CK, creatine kinase; GGT, gamma‐glutamyl transpeptidase; Neut, neutrophils; M, mature; PCV, packed cell volume; WBC, white blood cells.

NR, Not reported, Gaps indicate that the test was not performed.

During a recheck examination 4 days later (Day 5), a grade 2/6 left‐sided systolic heart murmur was heard; thoracic auscultation was otherwise unremarkable. The heart rate and respiratory rate were 120 beats per minute (bpm) and 28 breaths per minute (brpm), respectively. Hematological abnormalities included moderate regenerative anemia, marked leukocytosis and thrombocytosis (Table 1, Day 5). Given the increase in leukocytes, further investigations were performed. Urinalysis (cystocentesis‐derived sample) detected a urine specific gravity (USG) of 1.013, pyuria and gram negative bacilli, which cultured as *Escherichia coli*. Abdominal ultrasonography detected a diffusely hyperechoic liver, hyperechoic linear striations in both renal cortices, mild bilateral pyelectasia and hyperechoic speckles in the urinary bladder. The superficial margin of the mucosal layer of the duodenum was irregular with small concave defects. Pyelonephritis and duodenal ulceration were suspected. Prednisolone was reduced to 2.7 mg/kg/d and enrofloxacin (Baytril, Bayer Animal Health, Australia) was commenced at 10 mg/kg/d PO. Ciclosporin, azathioprine and omeprazole were continued. On Day 9, the dog had mild tachypnoea (36 brpm). Thoracic auscultation was unremarkable, and the previously appreciated heart murmur was not heard. Rectal temperature was 38.7°C, and the anemia was static (Table 1, Day 9). Prednisolone was reduced to 1.8 mg/kg/d and aspirin was discontinued.

The dog subsequently represented on Day 11 with marked tachypnoea (88 brpm), weakness, and multiple petechiations on the left and right caudoventral thorax and ventral abdomen. Diffusely increased bronchovesicular sounds, mild tachycardia (140 bpm), and mild hyperthermia (39.0°C) were present. The dog was hypoxic (SpO_2_ 88%). Respiratory tract infection or pulmonary thromboembolism (PTE) was suspected. Systemic inflammatory response syndrome or concurrent coagulopathy (eg, vasculitis), including disseminated intravascular coagulation, were also considered due to the new finding of spontaneous bruising with a normal platelet count.

**Figure 1:**
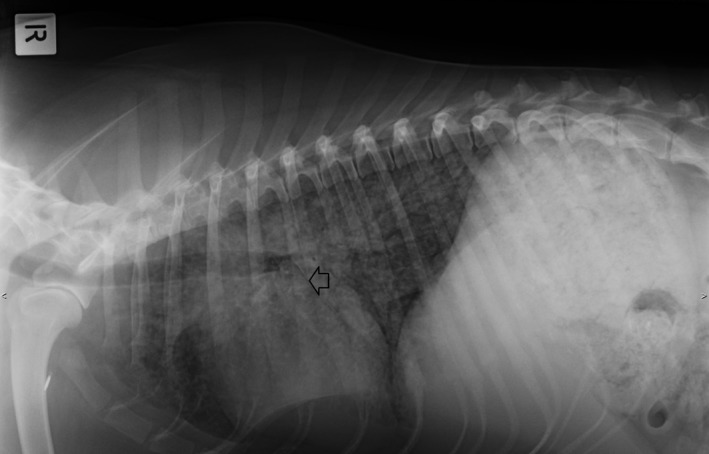
Right lateral thoracic radiograph obtained at Day 11. A diffuse, predominantly interstitial pattern is evident. Arrow shows fissure line

**Figure 2:**
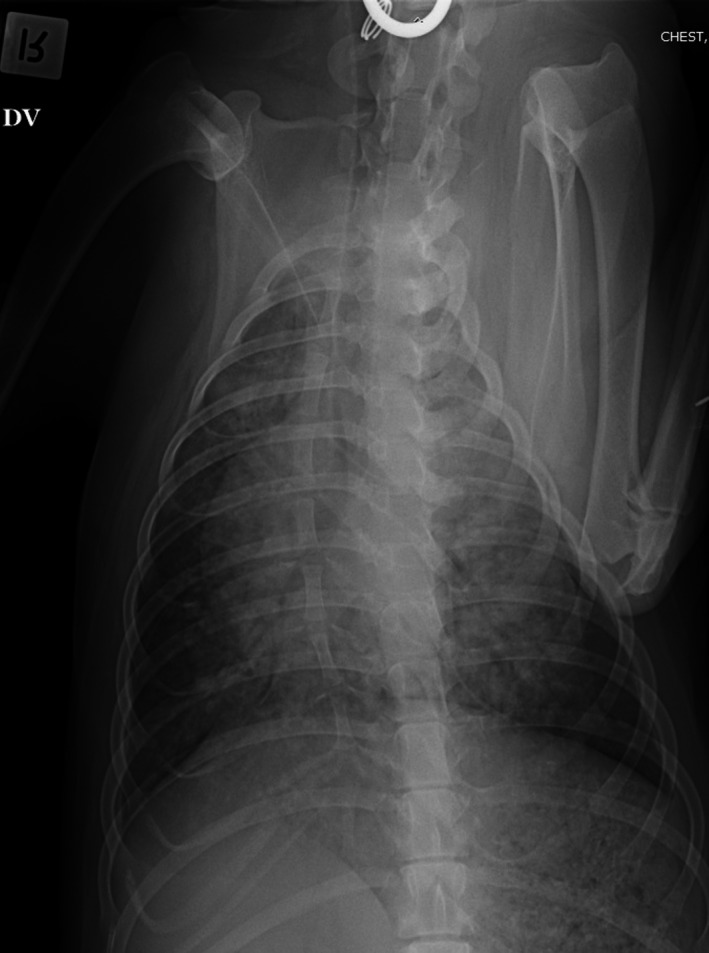
Dorsoventral thoracic radiograph obtained at Day 11

**Figure 3:**
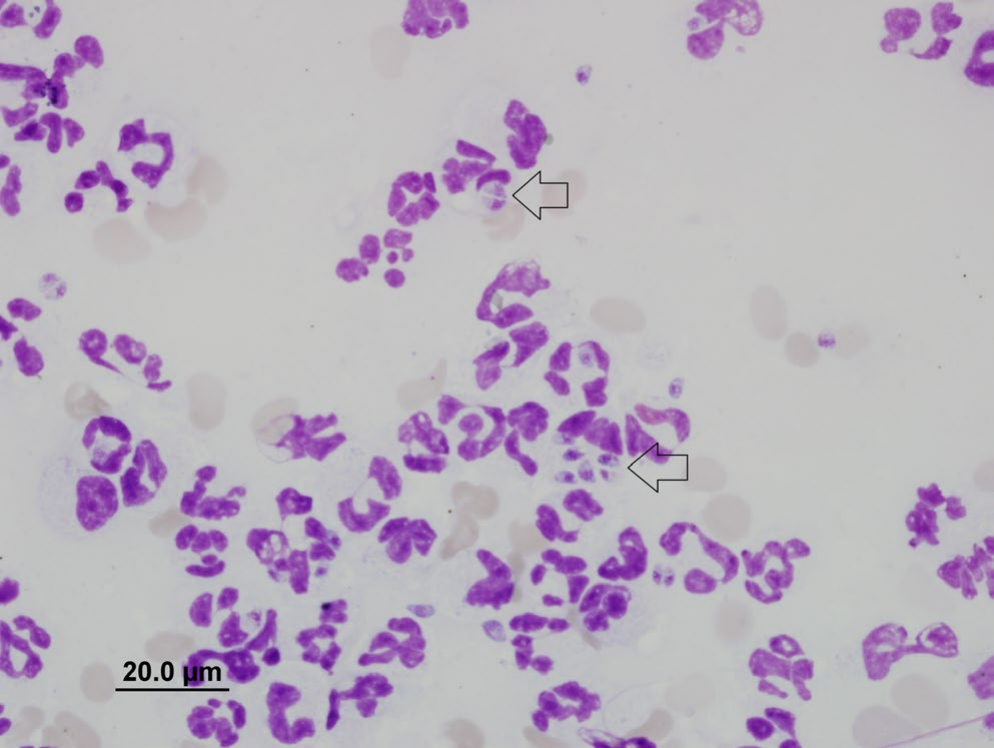
Cytology of BAL fluid obtained on Day 11. Cytospin and Wright‐Giemsa stain, magnification 100×. Numerous predominantly intracellular protozoal organisms are seen; some are illustrated by arrows

Nasal oxygen was given at 1 L/min via nasal prongs. Hematology and biochemistry were repeated (Table 1, Day 11). In house, APTT was 121 seconds (reference interval [RI] RI 72‐102), and partial thromboplastin time (PT) was 16 seconds (RI 11‐17), and buccal mucosal bleeding time (BMBT) was prolonged at 3 minutes (RI < 2 minutes). Thoracic radiographs showed a diffuse mixed (predominantly interstitial) pulmonary pattern in all lung lobes, thin pleural fissures consistent with a small volume pleural effusion and hepatomegaly (Figures 1 and 2). Repeat abdominal ultrasound findings were similar to the previous study. The respiratory distress worsened, hence the dog was intubated, mechanical ventilation was initiated to achieve better oxygenation, a blind bronchoalveolar lavage (BAL) was performed and arterial blood gas was measured shortly after establishment of ventilatory support. Cytology of the BAL fluid showed many degenerate neutrophils with predominantly intracellular protozoal organisms (Figure 3). Arterial blood gas analysis revealed marked hypercapnia, respiratory acidosis and persistent hypoxia (Table 2). Clindamycin (12.5 mg/kg, Dalacin C, Pfizer Australia Pty Ltd, Australia) was administered once slowly IV and ventilatory support was modulated to treat hypercapnia and hypoxia. Despite treatment, the dog deteriorated further and suffered cardiac arrest within 8 hours. On serum collected at the final presentation, *Neospora*IFAT was negative, and the *Toxoplasma* IFAT IgG titer was 1:128.

**Table 2:** Results of arterial blood gas analysis immediately after establishment of mechanical ventilation on Day 11

Parameter		Unit	Reference interval
FiO_2_	80	%	
pH	**7.18**		7.4 ± 0.5
pCO_2_	**66.7**	mm Hg	32‐43
pO_2_	86.3	mm Hg	80‐105
HCO_3_^−^	23.8	mmol/L	18‐26
Sat O_2_%	**94.9**	%	>98
SBE	−3.5		0 ± 4
Anion gap	13.7		12‐24
Lactate	**4.1**	mmol/L	<2

FiO_2_, fractional inspired oxygen concentration; HCO_3_^−^, Bicarbonate; pCO_2_, partial pressure of carbon dioxide in arterial blood; pO_2_, partial pressure of oxygen in arterial blood; Sat O_2_%, oxygen saturation of arterial blood; SBE, standard base excess.

Bold indicates outside of reference range.

**Figure 4:**
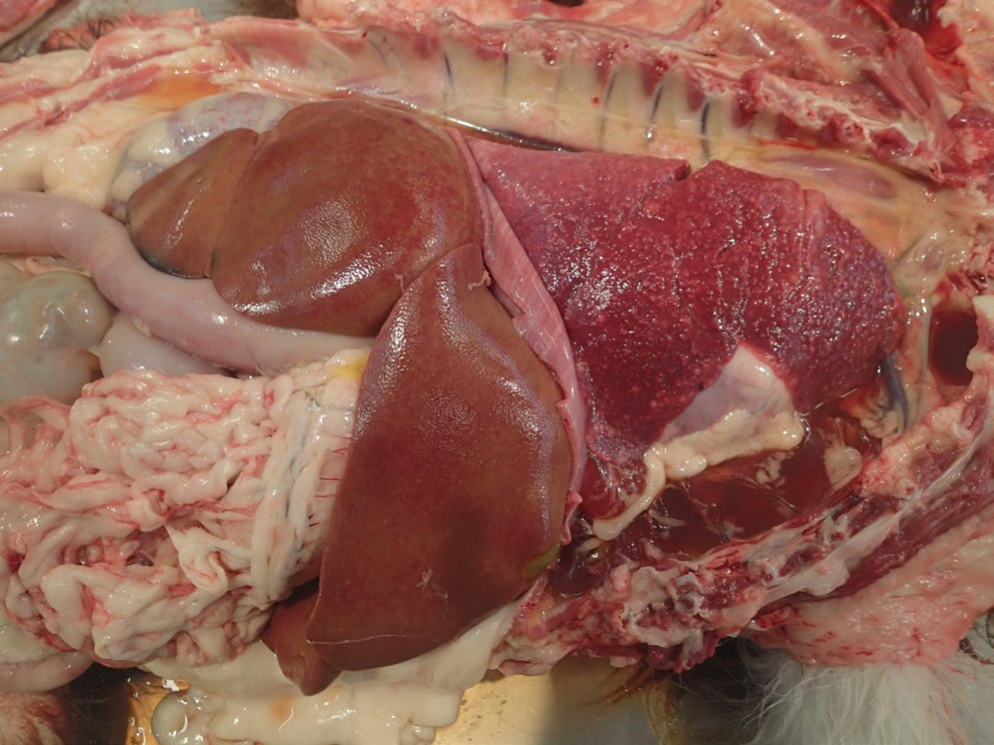
Gross postmortem image displaying lung nodules and consolidation as well as generalized liver swelling and pallor

**Figure 5:**
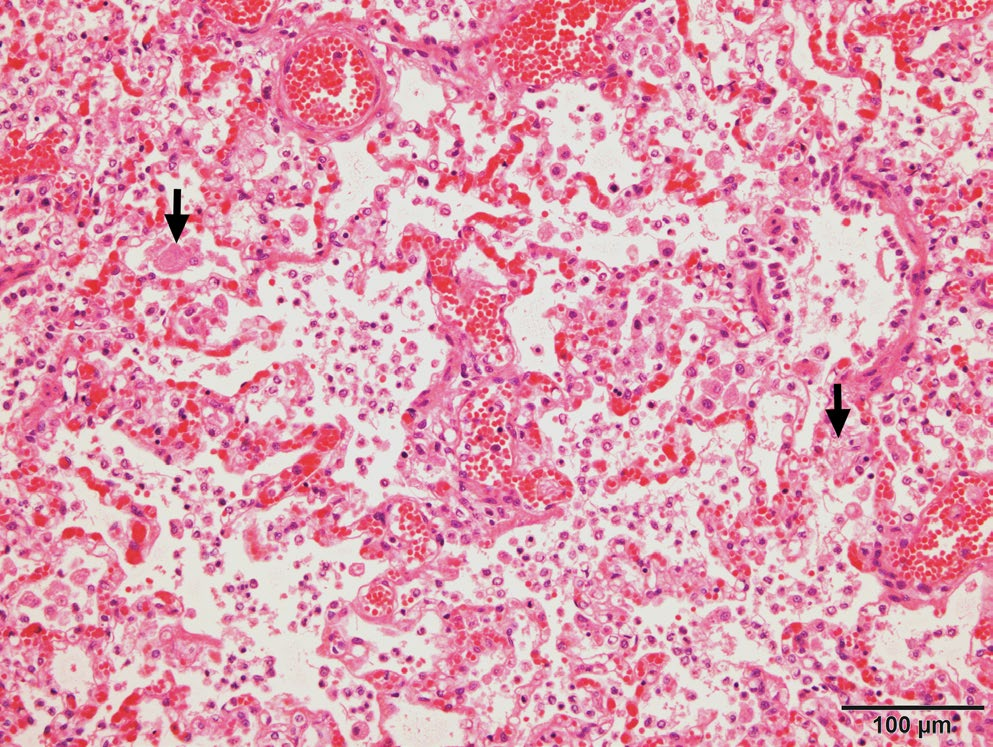
Photomicrograph of lung displaying marked infiltrate of macrophages within alveolar spaces and septa as well as fibrin aggregates (arrows). HE stain

**Figure 6:**
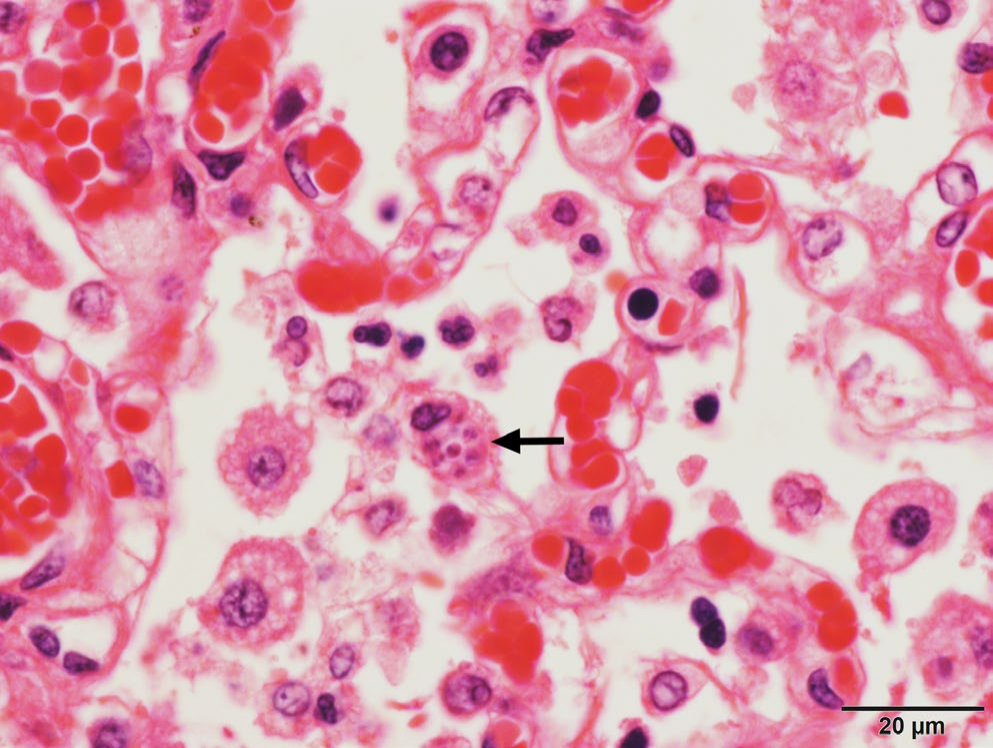
Photomicrograph of paired protozoal organisms within the cytoplasm of a macrophage (arrow). HE stain

Major gross findings on postmortem examination were a markedly swollen and pale tan liver overlayed by strands of fibrin, a small volume of red‐tinged serous pleural fluid and diffusely consolidated lungs with small white nodules distributed throughout all lobes (Figure 4). Wedge‐shaped regions of red discolouration were present in the kidneys, and irregular, dark red, depressed regions were also present in the spleen, consistent with infarction. Histopathological examination of tissues identified a marked neutrophilic and histiocytic interstitial pneumonia with multifocal infarction and fibrin exudation (Figure 5). Randomly distributed coalescing regions of coagulative necrosis containing fibrin aggregates were present in the hepatic parenchyma, as well as midzonal ballooning hepatocyte degeneration (consistent with steroid hepatopathy). Scattered foci of gliosis were present in the brain tissue, and lymphocyte populations in all lymphoid tissues were markedly depleted. Individual or small clusters of 1‐2 µm long ovoid to elongate protozoal organisms, which were often paired or with a bilobed nucleus (Figure 6), were present in the cytoplasm of cells in the lung, liver, lymph nodes, kidney, bladder and brain. Immunohistochemical testing identified *T gondii* antigen in small clusters and individually scattered diffusely throughout the lung parenchyma.

## 3 DISCUSSION

This report describes the occurrence of confirmed toxoplasmosis in a dog presenting with respiratory distress. The dog received prednisolone in combination with azathioprine and ciclosporin at the time of respiratory distress. These drugs are commonly used in dogs to treat immune‐mediated and inflammatory conditions, yet to date, systemic toxoplasmosis has only been reported in two dogs as a suggested iatrogenic complication of immunosuppression.^3^, ^11^ Including our case, none of the three dogs recovered from the disease. This emphasizes that, despite its apparent low prevalence, toxoplasmosis must be considered as a potentially fatal complication of immunosuppressive therapy in dogs.^8^, ^11^

Glucocorticoids remain the mainstay of treatment for immune‐mediated conditions such as IMHA. There is currently no solid evidence that supports the addition of other immunosuppressant drugs to standard treatment protocols of newly diagnosed dogs.^14^, ^15^ The dog in this case report had severe glucocorticoid‐refractory disease and showed intolerable side effects to glucocorticoids; azathioprine and ciclosporin were added in an attempt to address these concerns.

While it remains to be elucidated to what degree each individual immunosuppressive drug increases the risk of toxoplasmosis in dogs, it is likely that the combination of several immunosuppressive agents increases the risk and severity of toxoplasmosis by influencing host‐parasite interaction.^16^ The combination of specifically these three immunosuppressive drugs (ciclosporin, azathioprine, prednisolone) was found to result in unacceptably high rates of infection post‐renal transplantation, and immunosuppression was considered excessive.^17^

Cyclosporine and metabolites of azathioprine and glucocorticoids inhibit cell‐mediated immunity in a dose‐dependent manner,^18^ and several of the cats or dogs that have been reported to have fatal toxoplasmosis were treated with a combination of these drugs.^3^, ^11^, ^19^ Cyclosporine, in particular, may increase the risk of toxoplasmosis as it inhibits the production of several cytokines including interleukin‐2 and interferon‐γ (IFN‐γ), which markedly reduces T‐cell responses.^20^ A reduction of IFN‐γ may also reduce the anti‐parasitic ability of macrophages, natural killer cells and nonimmune cells, hence allowing intracellular growth, transmission and proliferation of *T gondii*.^21^ Systemic toxoplasmosis has been described after monotherapy with ciclosporin in cats^22^, ^23^ and localized toxoplasmosis may also occur in cats and dogs.^24^, ^25^ However, it must be noted that fatal toxoplasmosis in cyclosporine‐treated cats is uncommon^26^ and a direct inhibitory effect of ciclosporin metabolites on *T gondii* proliferation has also been described.^27^ Hence, the overall risk of development of toxoplasmosis in dogs treated specifically with ciclosporin remains unclear. The perceived risk of toxoplasmosis in dogs receiving only glucocorticoids is low, because glucocorticoids are commonly used to treat canine immune‐mediated conditions and so far, toxoplasmosis after glucocorticoid monotherapy has only been reported in one dog that concurrently suffered from lymphoma.^8^

Further study is required to assess whether *T gondii*serology before or during advanced immunosuppressive therapy is beneficial in regards to cost and outcome. In cats requiring suppression of cellular immunity, prior assessment of *Toxoplasma* serological status has been recommended to evaluate risk of toxoplasmosis.^22^ Exposure of seronegative cats to *T gondii*should be avoided by restricting hunting of small prey species and preventing access to sources containing the infectious stages of *T gondii*(eg, feces, water, raw meat).^22^ Similar strategies could be considered in dogs. However, many dogs may be seropositive due to previous exposure to *T gondii*.^4^, ^5^ Seropositivity before commencement of immunosuppressive treatment should not affect the initial therapeutic decisions, as a seropositive status per se may not predict recrudescence of toxoplasmosis or severity of disease.^1^ Furthermore, standard prophylactic use of antimicrobials such as clindamycin to prevent recrudescence of toxoplasmosis in seropositive dogs appears currently unwarranted. Prophylactic clindamycin use has been suggested in seropositive ciclosporin‐receiving cats 22 and prophylactic treatment with trimethoprim‐sulfonamide has been shown to reduce the risk of clinical toxoplasmosis in immunocompromised people.^28^ However, in immunosuppressed dogs, the prevalence of severe toxoplasmosis is apparently low. Furthermore, the aforementioned antimicrobials are unable to eradicate *T gondii* bradyzoites from tissues,^1^ and their use may be associated with adverse effects including paradoxical worsening of toxoplasmosis.^29^, ^30^, ^31^ Hence, further research is needed to assess whether prophylactic antimicrobials are beneficial in dogs.^1^

An increased IgM titer or a fourfold increase in IgG titers is highly suggestive of clinical toxoplasmosis.^1^ Thus, IgM serology after immunosuppressive therapy is begun, or paired IgG titers before and after the onset of immunosuppressive therapy could be considered in seropositive dogs. However, it is questionable whether onset of toxoplasmosis can be accurately predicted with such serological testing, because antibody production may be affected by immunosuppressive therapy.^26^, ^32^ Furthermore as also demonstrated in this report, low titers can be associated with severe clinical signs^1^ and peracute disease may remain undetected, particularly when only IgG titers are assessed.^33^

Knowledge of the individual's serological status before the onset of disease may allow differentiation between acute or latent infection. Recrudescence of latent infection is more common in immunocompromised people,^13^ but has been suspected in a number of cats and dogs.^3^, ^11^, ^19^, ^22^ The likelihood of recrudescence as a cause of infection in this dog is unknown, as the dog had consumed raw kangaroo prior to therapy. However, acute exposure to *T gondii* via feline feces or sporozoite‐contaminated water or through blood transfusion cannot be excluded as an alternative means of infection.

Demonstration of tachyzoites in the BAL fluid in combination with negative *Neospora* titers allowed definitive diagnosis of toxoplasmosis this case. Demonstation of tachyzoites in BAL fluid is usually diagnostic in cats with pulmonary toxoplasmosis.^22^, ^34^ A previous experimental study suggests that although cytology of BAL fluid is sensitive for the diagnosis of pulmonary toxoplasmosis, tachyzoites are not seen in every sample.^35^ *Toxoplasma gondii*is more readily detected by PCR of BAL fluid.^36^ When *T gondii* organisms or DNA cannot be demonstrated, serology in combination with clinical signs indicative of toxoplasmosis, exclusion of other causes, and positive response to treatment can be used to tentatively diagnose toxoplasmosis.^1^ This dog was relatively young for primary IMHA, and was not a breed known to be predisposed.^14^, ^37^ Screening for *Anaplasma* spp, *Ehrlichia* spp, *Babesia* spp or *Dirofilaria* spp was not performed as these agents are exotic to the area where the dog lived. Due to the rarity of clinical toxoplasmosis in immunocompetent dogs, routine exclusion of *T gondii*as a potential cause for secondary IMHA was not performed. However, it is possible that a secondary disease (such as toxoplasmosis or *E coli*urinary tract infection) caused or complicated IMHA in this dog, as there was limited control of anemia after 7 weeks of treatment. This case highlights that it may be beneficial to repeat or extend on previous tests if clinical signs persist, particularly if immunosuppressive therapy is to be escalated.

## 4 CONCLUSION

Toxoplasmosis as the cause of severe respiratory distress was confirmed in a dog that was treated with multiple immunosuppressive drugs at the onset of dyspnoea or immediately prior to it. The prevalence of clinical toxoplasmosis in immunosuppressed dogs remains to be determined; although at this stage it appears low despite high seroprevalence to *T gondii*.^4^, ^5^ However, toxoplasmosis is a potentially fatal complication in dogs receiving immunosuppressive therapy, particularly if multiple immunosuppressive drugs are used or high doses are chosen.^3^, ^11^ Further research is needed to assess whether certain immunosuppressive drugs, like ciclosporin, are associated with a higher risk of toxoplasmosis in dogs. Toxoplasmosis should be considered when signs of acute respiratory or hepatic disease develop, and diagnosis would rely on demonstration of organisms via cytology or PCR rather than a single time‐point serological assay.^36^ It remains to be elucidated whether IgG and IgM serology before and during immunosuppressive therapy is beneficial. Standard prophylactic treatment of seropositive dogs with antimicrobials such as clindamycin or trimethoprim‐sulfonamide appears unwarranted at this stage.

## CONFLICT OF INTEREST

The authors have no conflicts of interests to declare.

## AUTHOR CONTRIBUTION

Dr. AP: Made substantial contributions to conception, content, acquisition of data and design. Prof. CM: Involved in drafting the manuscript and revising it critically for important intellectual content. Dr. AS: Made contributions to content. Dr. TJ: Made substantial contributions to the conception, content, acquisition of data and design.
